# Complement Factor I Variants in Complement-Mediated Renal Diseases

**DOI:** 10.3389/fimmu.2022.866330

**Published:** 2022-05-10

**Authors:** Yuzhou Zhang, Renee X. Goodfellow, Nicolo Ghiringhelli Borsa, Hannah C. Dunlop, Stephen A. Presti, Nicole C. Meyer, Dingwu Shao, Sarah M. Roberts, Michael B. Jones, Gabriella R. Pitcher, Amanda O. Taylor, Carla M. Nester, Richard J. H. Smith

**Affiliations:** Molecular Otolaryngology and Renal Research Laboratories, University of Iowa, Iowa City, IA, United States

**Keywords:** complement, factor I, C3 glomerulopathy, atypical hemolytic uremic syndrome, C3 glomerulonephritis, dense deposit disease

## Abstract

C3 glomerulopathy (C3G) and atypical hemolytic uremic syndrome (aHUS) are two rare diseases caused by dysregulated activity of the alternative pathway of complement secondary to the presence of genetic and/or acquired factors. Complement factor I (FI) is a serine protease that downregulates complement activity in the fluid phase and/or on cell surfaces in conjunction with one of its cofactors, factor H (FH), complement receptor 1 (CR1/CD35), C4 binding protein (C4BP) or membrane cofactor protein (MCP/CD46). Because altered FI activity is causally related to the pathogenesis of C3G and aHUS, we sought to test functional activity of select *CFI* missense variants in these two patient cohorts. We identified 65 patients (16, C3G; 48, aHUS; 1 with both) with at least one rare variant in *CFI* (defined as a MAF < 0.1%). Eight C3G and eleven aHUS patients also carried rare variants in either another complement gene, *ADAMTS13* or *THBD*. We performed comprehensive complement analyses including biomarker profiling, pathway activity and autoantibody testing, and developed a novel FI functional assay, which we completed on 40 patients. Seventy-eight percent of rare *CFI* variants (31/40) were associated with FI protein levels below the 25^th^ percentile; in 22 cases, FI levels were below the lower limit of normal (type 1 variants). Of the remaining nine variants, which associated with normal FI levels, two variants reduced FI activity (type 2 variants). No patients carried currently known autoantibodies (including FH autoantibodies and nephritic factors). We noted that while rare variants in *CFI* predispose to complement-mediated diseases, phenotypes are strongly contingent on the associated genetic background. As a general rule, in isolation, a rare *CFI* variant most frequently leads to aHUS, with the co-inheritance of a *CD46* loss-of-function variant driving the onset of aHUS to the younger age group. In comparison, co-inheritance of a gain-of-function variant in *C3* alters the phenotype to C3G. Defects in *CFH* (variants or fusion genes) are seen with both C3G and aHUS. This variability underscores the complexity and multifactorial nature of these two complement-mediated renal diseases.

## Introduction

The complement cascade is the cornerstone of the innate defense system. It is the first line of defense against foreign and altered host cells, it activates and potentiates adaptive immunity, and it provides integrated crosstalk with other pathways including the coagulation pathway (1). Activation of complement is triggered *via* one of three pathways–the classical (CP), lectin (LP) and alternative (AP)–all of which converge on C3bBb, a C3 convertase that cleaves C3 to C3b, to amplify the complement response and initiate the terminal pathway with ultimate formation of the membrane-bound lytic unit membrane attack complex (C5b-9). The entire cascade is tightly regulated by several mechanisms involving soluble and membrane-bound regulators such as factor H (FH), factor I (FI) and membrane cofactor protein (MCP/CD46) (2).

**Figure 1:**
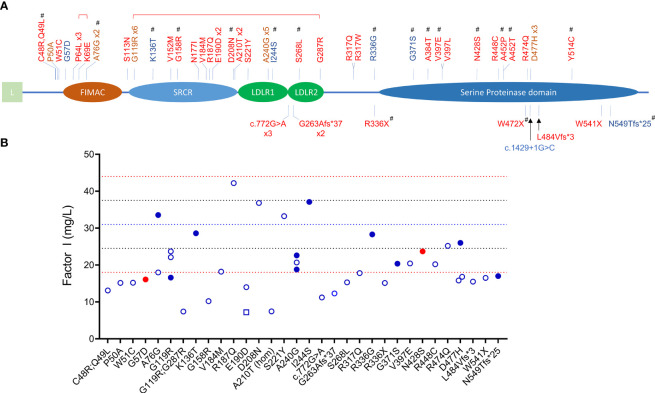
*CFI* variants identified in patients with C3G and aHUS. **(A)** Schematic view of factor I and the 45 rare variants (MAF < 0.1%) identified in 65 patients (red, aHUS; blue, C3G; brown, both C3G and aHUS). All variants were identified in heterozygosis except one homozygote (p.Ala210Thr) and two compound heterozygotes (brackets above variants indicate compound heterozygosity). Novel variants are marker with #. (L, leading sequence; FIMAC, Factor I Membrane Attack Complex; SRCR, Scavenger Receptor Cysteine-Rich; LDLR, Low-Density Lipoprotein Receptor). **(B)** FI levels in 40 patients (open circles, aHUS; filled blue circles, C3GN; filled red circles, DDD; open square, C3G/aHUS; dashed lines, reference values: red, upper and lower limits of normal; blue, percentile lines).

The primary function of FI is to downregulate complement activity by proteolytic inactivation of C3b to iC3b and C4b to iC4b in the presence of one of its four co-factors, FH, CR1, C4BP and MCP. FI-mediated regulation is especially important in controlling activity of the AP as this pathway is constitutively active. FI is synthesized by the liver as a 66 kDa single chain peptide that undergoes posttranslational glycosylation (adding 22 kDa of glycans) and protein cleavage to remove the signal sequence and four internal amino acids (RRKR, residues 336 to 339) to generate a mature protein with a heavy chain (50 kDa, Lys19–Ile335) and a light chain (38 kDa, Ile340–Val583) linked by a single disulfide bond (Cys255-Cys471) (3, 4). The five recognizable domains include a membrane-attack complex (FIMAC) domain, scavenger receptor cysteine-rich (SRCR) domain, two low-density lipoprotein receptor domains (LDLR1 and LDLR2) and, after a C-terminal linker on the heavy chain, the catalytic or serine protease (SP) domain with its catalytic triad His380-Asp429-Ser525 on the light chain (**Figure 1**) (5).

Deficiency of FI leads to unchecked AP amplification and consumptive deficiency of complement components, such as C3, with various phenotypic consequences (6). Complete absence of FI, which is rare, results in severe and recurrent infections, glomerulonephritis and/or autoimmune disease (7–11). Partial FI deficiencies, which are much more common, lead to dysregulated C3 convertase activity and predispose to several complement-mediated diseases such as atypical hemolytic uremic syndrome (aHUS) (12–15), age-related macular degeneration (AMD) (16–18), and occasionally, C3 glomerulopathy (C3G) (19–22).

C3G and aHUS are rare renal diseases caused by dysregulated complement activity of the AP typically driven by genetic and/or acquired factors. In C3G, uncontrolled complement activity in the fluid phase and glomerular microenvironment leads to a characteristic pattern of glomerular injury defined by the predominant deposition of C3 fragments for which the disease is named (23–25). Based on electron microscopy, two major subclasses of C3G are recognized–C3 glomerulonephritis (C3GN) and dense deposit disease (DDD). Classic clinical findings of both subtypes are hematuria and proteinuria (26). aHUS is a form of complement-mediated thrombotic microangiopathy (TMA) characterized by microangiopathic hemolytic anemia, consumptive thrombocytopenia, and multisystem end organ involvement primarily affecting the kidney (27). In aHUS, dysregulated complement activity primarily occurs on cell surfaces, leading to local prothrombotic states, especially in the kidney.

Here, we reported 45 variants in *CFI* (MAF < 0.1%) identified in 65 patients with either C3G or aHUS. We completed detailed studies in 40 patients that included a novel FI functional assay and identified both type 1 and type 2 *CFI* variants.

## Methods

### Patients

Our C3G and TMA registry (C3G, n = 1048; TMA, n = 1468) was searched for patients who were evaluated between 2009-’21 and carried a *CFI* variant with a minor allele frequency (MAF) < 0.1%. Biomaterials included DNA (all patients) isolated from peripheral blood using established methods (28), and serum and plasma (most patients) collected at the time of disease onset (acute flare) in patients with aHUS or during ongoing disease (chronic period) in patients with C3G using a standard protocol (29) and stored as one-time-use aliquots at -80°C. The study was approved by the Institutional Review Board of Carver College of Medicine at the University of Iowa.

### Genetic and Biomarker Analyses

Genetic analysis was performed using a targeted sequencing panel, which captures relevant complement genes as well as *THBD*, *ADAMTS13* and *DGKE* as we have described (30). In addition, the *CFH-CFHR* and *CFI* genomic regions were screened for copy number variation by multiplex ligation-dependent probe amplification (MLPA) using the MRC Holland SALSA kit and in-house designed probes.

Biomarker/functional testing was performed using a customized panel that includes ELISAs, radial immunodiffusion (RID), immunofixation electrophoresis (IFE), and hemolytic-based assays (29). Specifically, CP and AP activities were assessed using Quidel (San Diego, CA) and Wieslab kits (SVAR, Malmö, Sweden). Fluid-phase activity was also tested using IFE on a SPIFE machine (Helena Laboratories, Beaumont, TX). Serum levels of C3, C5, properdin, FH, FI and complement activation products C3c, Ba, Bb, sC5b-9 were measured using ELISA kits (Hycult Biotech, Wayne, PA or Quidel, San Diego, CA). C4, FB and FI were run by RID (The Binding Site, San Diego, CA). FH and FB autoantibody assays were done by ELISA against purified proteins FH and FB, respectively (Complement Tech, Tyler, TX). C3-, C4-, and C5-nephritic factors were detected by cell-based hemolytic methods (31).

### Factor I Co-Factor Activity

To assess FI co-factor activity, we developed a novel FI functional assay using C3b-decorated sheep erythrocytes (C3b-ShE) prepared as previously described (32). To minimize the effect of endogenous FH, patient serum or plasma was diluted 1:32 in GVB-Mg^2+^ buffer (16). To assess FI function, 50 µL of diluted serum/plasma, 50 µL of C3b-ShE (1x10^9^/mL) and 100 µL of co-factors were added to 200 µL of GVB-EDTA buffer. The co-factors [FH (Complement Tech, Tyler, TX), sCR1 (Celldex Therapeutics, New Haven, CT) or sMCP (Alexion Pharmaceuticals, Boston, MA)] were assayed at final concentrations of 50nM, 25nM and 50nM, respectively. The resulting mixture was incubated at 37°C for 15 minutes. After 3 washes, cells were resuspended in 50 µL of GVB-Mg^2+^ buffer and remaining C3b on ShE was titrated out by excess FB (5x of the amount at Z = 1) and FD (0.3 μg) in GVB-Mg^2+^ buffer to form C3 convertase at 30°C over a 5-minute period; the reaction was stopped by adding 300 µL of GVB-EDTA buffer. 50 μL of the mixture was transferred to an empty 96-well plate and hemolysis was induced by adding 50 μL of rat EDTA serum diluted (1:10) in GVB-EDTA buffer (as a source of C5-C9). After centrifugation at 1000 x g, cell-free supernatant was transferred to a flat bottom 96-well plate and absorbance was read at OD415. The percentage of hemolysis in each well was calculated using hypotonic lysis induced by water as 100%.

### Western Blot

Serum or plasma (1:80 diluted) in Laemmli buffer with or without reducing reagent was separated on 4-15% polyacrylamide gels (Bio-Rad, Hercules, CA). After transferring, FI was visualized using rabbit monoclonal antibody to FI (ab278524, Abcam, Waltham, MA) followed by a secondary incubation and chemiluminescence.

### Statistics

Statistical analysis was performed using the Student’s *t*-test for two group comparisons, Fisher’s exact test for contingency tables, and Pearson correlation to measure linear relationships between variables using GraphPad Prism 8.2 (GraphPad Software, San Diego, CA). Error bars represent means ± SD. The number of samples and number of experimental repetitions are indicated in the figure legends. *P* < 0.05 was considered significant.

## Results

### Patient Cohorts and Rare Variants in *CFI*

**Figure 2:**
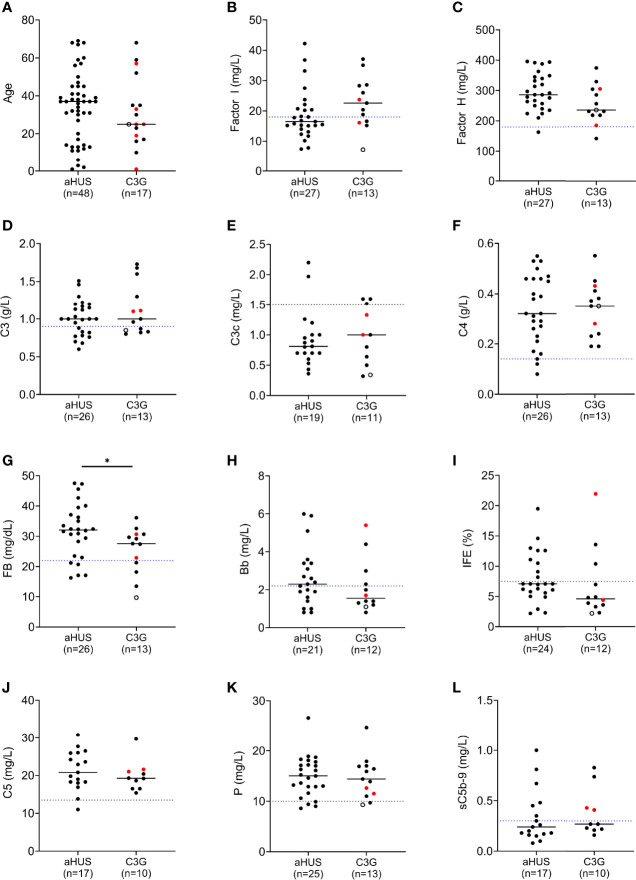
Complement biomarker profiling in aHUS and C3G patients. **(A)** Age distribution. **(B)** Factor I levels. **(C)** Factor H levels. **(D–H)** Biomarkers for alternative pathway (AP) activity including C3 and factor B, and associated activation products C3c and Bb. **(I)** AP fluid-phase activity by immunofixation eletrophoresis. **(J–L)** Biomarkers for terminal pathway activity including C5, properdin and soluble terminal complement complex (sC5b-9). (left column, aHUS; right column-red dots, DDD patients; black dots, C3GN patients; open circle, C3GN/aHUS patient; dashed line, lower limit of normal value for each assay; patients on eculizumab excluded from **(J, L)**; *P < 0.05).

Sixty-five unrelated patients (sixteen C3G [ten, C3GN; six, DDD]; 48 aHUS; one C3G/aHUS) with rare variants in *CFI* were identified in our registry (**Figure 1**), which represents significant enrichment in our aHUS cohort as compared to the prevalence of rare variants with MAF < 0.1% reported in gnomAD (1.77% of 141433 individuals in gnomAD vs 1.62% of 1048 C3G patients (*P* > 0.05) and vs 3.34% of 1468 TMA patients (*P* < 0.0001)) (33). In addition, the prevalence of rare variants is significantly higher in aHUS as compared to C3G (*P* = 0.0078). Disease occurred at any age in both cohorts (median age, 27.5 vs 37, respectively), although more aHUS patients were diagnosed in early childhood (age < 5, six aHUS vs one C3G). In the aHUS cohort, there were also more females (female/male, 35/13), while in the C3G cohort, both genders were equally affected (male/female, 9/8) (**Figure 2A**). Demographic data and basic clinical information are shown in **Table 1**.

**Table 1:** Patient demographics.

Characteristics	C3G		aHUS
	C3GN	DDD	
Patients, n	11	6	48
Sex (Male/Female)	7/4	2/4	13/35
Age, yr (median, quartile range)	30, 20-42	25, 21-31	37, 16-45
Onset, median age (yr, quartile range)	25, 14-37	17, 12-23	30, 10-38
Ethnicity			
Caucasian (n, %)	9 (82%)	6 (100%)	36 (75%)
Non-Caucasian (n, %)	2 (18%)	0 (0%)	12 (25%)
Proteinuria (n/data avail, %)	11/11 (100%)	6/6 (100%)	11/14 (79%)
Hematuria (n/data avail, %)	8/11 (73%)	2/2 (100%)	13/20 (65%)
Outcome and treatments			
ESRD, n	3	2	24
Transplantation, n	1	0	7
Failed transplant, n	1	0	2
complement C5 inhibition, n	4	0	15

Null alleles were found in nine of 48 (19%) patients with aHUS and two of 15 (13%) patients with C3G (**Tables 2**, **3**). Three aHUS patients (#27, #28, #29) carried the same splice-region variant (c.772G>A, p.Ala258Thr; the last nucleotide of exon 5), which results in exon 5 skipping (34). Three patients (#35, #46, #48) carried nonsense mutations (p.Arg336Ter, p.Trp472Ter and p.Trp541Ter) and three patients (#31, #32, #47) had micro-deletions that result in frame shifts (two with c.786delA and one with c.1450_1454del). In the C3G cohort, two null alleles were identified–a microdeletion (c.1646delA; #58, C3GN) and a splice site variant (c.1429+1G>C; #64, DDD). Western blot confirmed the absence of detectable circulating truncated proteins in all patients with null alleles, suggesting that the transcribed mutant message undergoes nonsense-mediated decay. We also confirmed non-expression of the c.1429+1G>C allele on a western blot in a homozygote patient we are following for recurrent infections.

**Table 2:** Demographic and genetic data in patients with aHUS.

ID	Age	Onset	Sex	DNA	Protein	Domain	MAF (gnomAD)	CADD	Other genes (including *ADAMTS13*, *THBD*, terminal pathway genes)	*CFHR3-1-4-2-5*
1	37	36	M	c.142T>C; c.146A>T**	p.Cys48Arg; p.Gln49Leu**		Novel	26.4 24.1		wildtype
2	67	65	F	c.148C>G	p.Pro50Ala		9.6E-05	24.8		wildtype
3	41	32	F	c.153G>T	p.Trp51Cys		Novel	28		del(*CFHR3-1*)
4	6	3	F	c.191C>T	p.Pro64Leu	FIMAC	2.3E-04	27.2	*CFH* c.3553G>C, p.A1185P *CD46* c.191G>A, p.C64Y	wildtype
5	13	5	F	c.191C>T	p.Pro64Leu	FIMAC	2.3E-04	27.2		wildtype
6	50	43	F	c.191C>T; c.205A>G^	p.Pro64Leu; p.Lys69Glu^	FIMAC	2.3E-04 2.4E-05	27.2 25.4		del(*CFHR3-1*)
7	11	3	F	c.227C>G	p.Ala76Gly	FIMAC	Novel	15.5	*CD46* c.350_351dupAC^#^	wildtype
8	37	31	M	c.338G>A	p.Ser113Asn		2.1E-05	8.8		del(*CFHR3-1*)
9	1	1	F	c.355G>A	p.Gly119Arg	SRCR	4.2E-04	23	*CD46* c.565T>G, p.Y189D	wildtype
10	34	22	F	c.355G>A; c.859G>A^	p.Gly119Arg; p.Gly287Arg^	SRCR	4.2E-04; 4.6E-05	23; 23.2		wildtype
11	45	35	F	c.355G>A	p.Gly119Arg	SRCR	4.2E-04	23		Not done
12	47	40	F	c.355G>A	p.Gly119Arg	SRCR	4.2E-04	23		wildtype
13	45	38	F	c.355G>A	p.Gly119Arg	SRCR	4.2E-04	23		del(*CFHR3-1*)
14	32	20	F	c.454G>A	p.Val152Met	SRCR	4.2E-05	27.8		wildtype
15	13	5	M	c.472G>A	p.Gly158Arg	SRCR	Novel	28.2		Not done
16	27	20	F	c.530A>T	p.Asn177Ile	SRCR	6.0E-05	16.8		del(*CFHR3-1*)
17	31	31	F	c.550G>A	p.Val184Met	SRCR	Novel	22	*ADAMTS13* c.2753T>C, p.L918P	del(*CFHR3-1*)
18	60	52	F	c.560G>A	p.Arg187Gln	SRCR	7.8E-05	16.5		del(*CFHR3-1*)
19	37	37	F	c.570G>T	p.Glu190Asp	SRCR	8.0E-06	24.1		wildtype
20	2	1	F	c.622G>A	p.Asp208Asn	SRCR	4.0E-06	8.4		wildtype
21	30	27	F	c.628G>A^#^	p.Ala210Thr^#^	SRCR	1.2E-05	23.7		del(*CFHR3-1*)^#^
22	45	38	M	c.628G>A	p.Ala210Thr	SRCR	1.2E-05	23.7		del(*CFHR3-1*)^#^
23	68	62	M	c.662C>A	p.Ser221Tyr	LDLR1	4.0E-06	9.3		del(*CFHR3-1*)
24	31	22	F	c.719C>G	p.Ala240Gly	LDLR1	2.5E-04	23.8		wildtype
25	20	11	F	c.719C>G	p.Ala240Gly	LDLR1	2.5E-04	23.8		del(*CFHR3-1*)
26	39	33	M	c.719C>G	p.Ala240Gly	LDLR1	2.5E-04	23.8		wildtype
27	56	55	F	c.772G>A	p.Ala258Thr	LDLR2	1.2E-04	34		Not done
28	38	30	M	c.772G>A	p.Ala258Thr	LDLR2	1.2E-04	34		wildtype
29	39	32	F	c.772G>A	p.Ala258Thr	LDLR2	1.2E-04	34		wildtype
30	14	6	F	c.803C>T	p.Ser268Leu	LDLR2	Novel	26.4	*CD46* c.768C>A, p.C256X^#^	wildtype
31	57	45	M	c.786delA	p.Gly263Alafs*37	LDLR2	2.0E-05		*C9* c.1030A>G, p.T344A	wildtype
32	13	4	F	c.786delA	p.Gly263Alafs*37	LDLR2	2.0E-05			del(*CFHR3-1*)
33	31	26	M	c.950G>A	p.Arg317Gln		2.1E-05	18.6		wildtype
34	37	34	F	c.949C>T	p.Arg317Trp		8.5E-05	14.9		wildtype
35	37	29	F	c.1006C>T	p.Arg336Ter		Novel	35		wildtype
36	12	1	M	c.1150G>A	p.Ala384Thr	SP	7.8E-05	3.7		Not done
37	59	51	M	c.1190T>A	p.Val397Glu	SP	Novel	17.1		wildtype
38	36	30	F	c.1189G>T	p.Val397Leu	SP	Novel	0.001	*CFH* c.575G>A, p.C192Y	wildtype
39	68	60	F	c.1342C>T	p.Arg448Cys	SP	7.2E-05	11.1		del(*CFHR3-1*)
40	11	8	F	c.1354G>C	p.Ala452Pro	SP	3.2E-05	25.7	*THBD* c.1465G>T, p.D489Y	wildtype
41	38	36	F	c.1354G>A	p.Ala452Thr	SP	1.2E-05	25.1		wildtype
42	41	33	F	c.1421G>A	p.Arg474Gln	SP	4.8E-05	22.1		del(*CFHR3-1*)
43	14	5	F	c.1429G>C	p.Asp477His	SP	2.0E-05	35		del(*CFHR3-1*)
44	3	2	F	c.1429G>C	p.Asp477His	SP	2.0E-05	35		wildtype
45	17	11	M	c.1541A>G	p.Tyr514Cys	SP	4.0E-06	15.8	*CD46* c.350_351dupAC	Not done
46	32	24	M	c.1415G>A	p.Trp472Ter	SP	Novel	41		*CFH-CFHR1* fusion
47	69	69	F	c.1450_1454del	p.Leu484Valfs*3	SP	2.8E-05		*C3* c.1898A>G, p.K633R	del(*CFHR3-1*)^#^
48	40	40	F	c.1622G>A	p.Trp541Ter	SP	Novel	42		wildtype

All variants (including MLPA results) identified are heterozygotes except:

^#^homozygote.

^compound heterozygote.

**on same allele.

**Table 3:** Demographic and genetic data in patients with C3G.

ID	Age	Onset	Sex	DNA	Protein	Domain	MAF(gnomAD)	CADD	Other genes (including *ADAMTS13*, *THBD*, terminal pathway genes)	*CFHR3-1-4-2-5*
C3GN										
49	30	25	F	c.227C>G	p.Ala76Gly	FIMAC	Novel	15.5	*C3* c.2203C>T, p.R735W	wildtype
50	68	67	M	c.355G>A	p.Gly119Arg	SRCR	4.2E-04	23	*CFH* c.3628C>T, R1210C	wildtype
51	35	28	F	c. 407A>C	p.Lys136Thr	SRCR	Novel	4.9		wildtype
52	59	47	M	c.719C>G	p.Ala240Gly	LDLR1	2.5E-04	23.8	*CFH* c.790+1 G>A	del(*CFHR3-1*)
53	16	13	F	c.719C>G	p.Ala240Gly	LDLR1	2.5E-04	23.8	*C3* c.2203C>T, p.R735W	wildtype
54	23	15	F	c.731T>G	p.Ile244Ser	LDLR1	3.6E-05	23		wildtype
55	10	7	M	c.1006C>G	p.Arg336Gly		5.2E-05	22.9		wildtype
56	35	28	M	c.1111G>A	p.Gly371Ser	SP	Novel	31		wildtype
57	52	46	M	c.1429G>C	p.Asp477His	SP	2.0E-05	35		Not done
58	17	12	M	c.1646delA	p.Asn549Thrfs*25	SP	Novel			del(*CFHR3-1*)
DDD										
59	25	12	F	c.148C>G	p.Pro50Ala		9.6E-05	24.8		wildtype
60	25	23	F	c.170G>A	p.Gly57Asp	FIMAC	4.0E-06	23.1		wildtype
61	1	1	M	c.191C>T	p.Pro64Leu	FIMAC	2.3E-04	27.2	*C3* c.481C>T, p.R161W	del(*CFHR3-1*)
62	19	12	F	c.355G>A	p.Gly119Arg	SRCR	4.2E-04	23	*C3* c.4594C>T, p.R1532W	del(*CFHR3-1*)
63	57	57	F	c.1283A>G	p.Asn428Ser	SP	Novel	23.4	*CFH* c.1056T>A, p.Y352X *C6* c.1375A>T, p.K459X	wildtype
64	33	22	M	c.1429+1G>C	Exon skipping	SP	2.8E-05	33	*C9* c.1042delA	wildtype
C3GN/aHUS										
65	25	19	M	c.570G>T	p.Glu190Asp	SRCR	8.0E-06	24.1		del(*CFHR3-1*)

All variants (including MLPA results) identified are heterozygotes.

Most variants (64/65) were found in heterozygosis, although we identified one aHUS patient homozygous for p.Ala210Thr (#21) and two who were compound heterozygotes (p.Pro64Leu/p.Lys69Glu, #6; p.Gly119Arg/p.Gly287Arg, #10). One aHUS patient carried two variants on the same allele (p.Cys48Arg/p.Gln49Leu, #1) (**Table 2**). No C3G patients carried a second rare variant in *CFI* and no large genomic deletions in the *CFI* region were identified (**Tables 3**).

### Additional Genetic and Acquired Drivers

Genetic variants (pathogenic or likely pathogenic, MAF < 0.1%) in other complement or TMA genes were identified in ten patients with aHUS and eight with C3G. One aHUS patient (#46) carried a *CFH*-*CFHR1* fusion gene identified by MLPA (**Table 2**). In addition, two DDD patients carried a null allele in a terminal pathway gene (#63, #64) (**Table 3**).

Homozygous deletions of *CFHR3-CHR1* were found in 3/60 (5%) patients, however none was positive for factor H autoantibodies (FHAAs). All patients were also negative for FBAAs and all C3G patients were negative for C3-, C4- and C5-nephritic factors.

### Factor I Levels and Complement Dysregulation

FI levels were measured in 40 patients (12 C3G; 27 aHUS and 1 C3G/aHUS) (**Figure 2B** and **Supplementary Tables 1**, **2**). In 31 of 40 (78%) patients, these levels were in the lowest quartile of the normal reference range (24.5 mg/L, normal reference mean = 31, standard deviation = 6.5). The lowest FI levels were found in two aHUS patients with biallelic variants. Excluding these two patients, FI levels were higher in C3G patients as compared to aHUS patients, however the difference was not statistically significant (median C3G vs aHUS, 24 vs 19.4, *P* = 0.09). FH levels showed the reverse trend and tended to be lower in C3G patients, although this comparison also did not reach statistical significance (**Figure 2C**, *P* = 0.08).

Several serum complement biomarkers were abnormal, including C3, which was low in four of twelve (33%) C3G and nine of 27 (33%) aHUS patients (**Figure 2D**), consistent with increased C3 convertase activity. In both cohorts, elevation in C3c occurred in a few patients while C4 was normal in most patients (**Figures 2E, F**). FB was low in three of twelve (25%) patients with C3G and five of 26 (19%) aHUS patients (**Figure 2G**), while Bb was high in four of eleven (36%) C3G and twelve of 21 (57%) aHUS patients (**Figure 2H**). Overall, the levels of FI showed moderate correlation with plasma C3 and factor B levels (Pearson’s *r* correlation = 0.39 and 0.36; *P* = 0.014 and 0.026, respectively). These findings also reflect increased convertase activity associated with a decrease in FI. Ba was elevated in most patients (**Supplementary Tables 1**, **2**) however this biomarker does not accurately reflect complement dysregulation but rather the degree of renal injury (35, 36). The positive IFE in three of eleven (27%) C3G and nine of 24 (38%) aHUS patients recapitulated dysregulated upstream complement activity (**Figure 2I**). Of terminal pathway biomarkers (**Figures 2J–L**), sC5b-9 was elevated in six of seventeen (35%) aHUS and four of ten (40%) C3G patients not on Eculizumab (**Figure 2L**).

### Cell-Based FI Functional Assay

**Figure 3:**
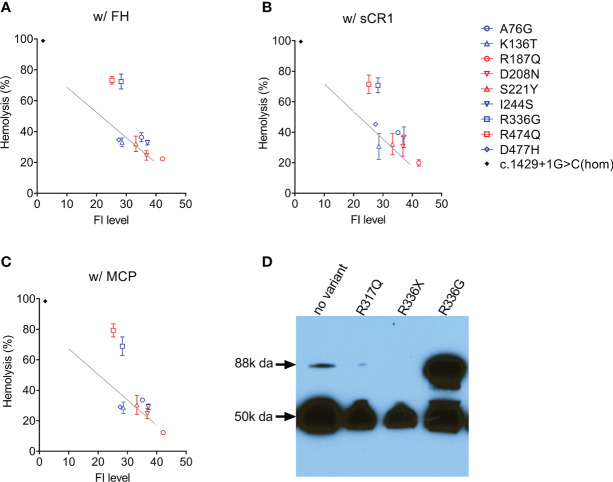
Assessing FI function. In the presence of a cofactor **(A)** FH 50 nM; **(B)** sCR1 25nM; **(C)** MCP 50nM, C3b cleavage activity by FI in patient serum (diluted 1:32) is measured using C3b-decorated sheep erythrocytes. Non-cleaved C3b is developed by the addition of both FB/FD to form C3 convertase and rat-EDTA serum as a source of C5-C9. Percent hemolysis is calculated and plotted as a function of FI concentration determined by ELISA. The grey line in each figure serves as a normal reference line calculated from a normal sample (FI concentration, 40 mg/L) with serial dilutions. Serum from a *CFI* c.1429+1G>C homozygote (FI is undetectable) serves as a positive control (black dot). **(D)** Circulating FI by a Western blot. Patient sample (1:80 diluted in PBS) with reducing reagent separated on a 4-15% polyacrylamide gel and transferred. FI was visualized by an antibody specific to the heavy chain (50k Da). *CFI* p.Arg336Gly results in an unprocessed single chain (88k Da).

In nine patients [four aHUS patients (p.Arg187Gln, p.Asp208Asn, p.Ser221Tyr, p.Arg474Gln); five C3G patients (p.Ala76Gly, p.Lys136Thr, p.Ile244Ser, p.Arg336Gly, p.Asp477His)], FI levels were above the lowest quartile and in these patients, we evaluated FI co-factor activity. Two type 2 variants were identified–p.Arg474Gln and p.Arg336Gly–both of which reduced C3b cleavage activity with all three cofactors (FH, sCR1 and MCP) (**Figures 3A–C**). Western blotting showed that p.Arg336Gly circulates as unprocessed single chain (**Figure 3D**). No functional impairment of FI activity was detectable with the other variants.

## Discussion

Sixty-five unrelated patients [sixteen C3G (ten, C3GN; six, DDD); 48 aHUS; one C3G/aHUS] with rare variants in *CFI* were identified in our registry, which represents significant enrichment in our aHUS cohort as compared to the prevalence of rare *CFI* variants reported in gnomAD (1.77% of 141433 individuals in gnomAD vs 1.62% of 1048 C3G patients (*P* > 0.05) and vs 3.34% of 1468 TMA patients (*P* < 0.0001)) (33). Eighteen of these variants are novel and three (p.Val184Met, p.Arg448Cys, p.Trp541Ter) are reported for the first time in an aHUS cohort [previously associated only with AMD (18, 37–39)]. In 40 patients, we completed serological and functional studies to facilitate the classification of these variants based on ACMG guidelines.

Low FI levels have a variety of phenotypic consequences with the most extreme, complete CFI deficiency, leading to uncontrolled AP activity and C3 consumption (7). As a consequence of C3 consumption, severe and recurrent pyogenic infections with encapsulated organisms such as *Streptococcus pneumoniae*, *Haemophilus influenzae* and *Neisseria meningitidis* develop, driven by defective opsonization, immune adherence and phagocytosis (40). Whilst we have not seen complete FI deficiency in our C3G and aHUS cohorts, we have identified several patients with low or borderline low FI levels (16-24.5 mg/L, normal range 18 – 44) with the presenting phenotype typically driven by other acquired or genetic factors. For example, in isolation a rare *CFI* variant most frequently leads to aHUS, however the co-inheritance of a *CD46* loss-of-function pathogenic variant drives the onset of aHUS to the younger age group (#4, #7, #9, #30, #45) while a gain-of-function pathogenic variant in *C3* alters the phenotype to C3G (4 of 15 *vs* 1 of 48, C3G *vs* aHUS, *P* = 0.0097). Defects in *CFH* (variants or fusion genes) are seen with both C3G and aHUS (3 of 15 *vs* 2 of 48, C3G *vs* aHUS, *P* = 0.0828).

Autoantibodies to complement components (such as FHAAs, FBAAs, C3-, C4-, C5-nephritic factors) also play an important role in the pathogenesis of C3G and aHUS (23, 41), and while none of the patients in this study was co-positive for autoantibodies, we did not consider this finding surprising. With respect to aHUS, the prevalence of FHAAs is generally low, and when identified, they are typically seen in the pre-teenage years in children who are homozygous for the deletion of *CFHR3-CFHR1*. This genotype was identified only three times in our aHUS cohort, all in adults. With respect to C3G, about 60% of patients are positive for genetic mutations and/or autoantibodies as drivers of disease (19, 29). Of this 60%, ~10% are co-positive for both genetic drivers and autoantibodies, ~70% have only autoantibodies, and ~20% have only genetic variants. In our C3G cohort, half had additional genetic variants contributing to the complement dysregulation.

In 27 patients in our aHUS cohort, we measured FI levels and identified 18 type 1 variants (low expression) and five variants with limited expression (within 1^st^ quartile, **Figure 1B**). The remaining 4 variants–p.Arg187Gln, p.Asp208Asn, p.Ser221Tyr and p.Arg474Gln–were associated with normal FI levels (above the lowest quartile). Of these, p.Asp208Asn is a novel variant, while p.Arg187Gln, p.Ser221Tyr and p.Arg474Gln have been reported. Although no data are provided for p.Arg187Gln, p.Ser221Tyr was associated with normal FI levels in two AMD studies (16, 18). p.Arg474Gln has also been reported in AMD cohorts with normal FI levels (18, 42, 43) and in an aHUS cohort with low FI levels, although the aHUS patient also carried another type 1 variant p.Ala258Thr (44). Our functional data show that p.Arg474Gln affects FI cofactor activity with all three cofactors (FH, MCP, sCR1) (**Figure 3**), making p.Arg474Gln a type 2 pathogenic variant. No functional defects were observed with p.Arg187Gln, p.Asp208Asn and p.Ser221Tyr, and based on these data, these three variants should be classified as likely benign.

Low FI levels (< 25%) were found in six C3GN patients and one DDD patient (#60). Of the six C3GN patients, two *CFI* variants were identified in each of two patients (p.Gly119Arg, #50 and #62; p.Ala240Gly, #52 and #53), with the remaining two patients carrying truncating variants (p.Asn549Thrfs*25, #58; c.1429+1G>C, #64). Interestingly, p.Gly119Arg and p.Ala240Gly were also identified in aHUS patients (p.Gly119Arg, #9, #10, #11, #12, #13; p.Ala240Gly, #24, #25, #26) and are well-documented type 1 variants (18, 21, 45–48). p.Gly119Arg and p.Ala240Gly are also present in the general population with the highest minor allele frequencies in Europeans (MAF = 0.000852 and 0.000093, respectively). These data suggest that carrying p.Gly119Arg or p.Ala240Gly is a risk factor for complement-mediated disease depending, in part, on the expressivity of the other *CFI* allele and/or the presence of mutations in other genes. For example, one aHUS patient (#10) had extremely low FI levels as a result of compound heterozygosis (p.Gly119Arg and p.Gly287Arg on opposite alleles), while all C3G patients carrying either the p.Gly119Arg allele or the p.Ala240Gly allele had a second pathogenic or likely pathogenic variant in *CFH* or *C3* (#50, #52, #53, #62; **Table 3**).

Similarly, p.Pro64Leu, a variant that has been documented in AMD patients with low FI expression (16, 18), was identified in three aHUS patients (one *CFI* compound het) and one DDD patient (#4, #5, #6 and #61). The DDD patient also carried a gain-of-functions variant in *C3*, p.Arg161Trp, which has been shown to increase C3b affinity for factor B and reduce binding to MCP, although FH-mediated regulation is unchanged (14, 49, 50).

With two other variants [p.Ala76Gly (novel), p.Asp477His] identified in both C3GN and aHUS patients (#49 *vs* #7 and #57 *vs* #43 and #44, C3G *vs* aHUS), we noticed much higher FI levels in C3G as compared to aHUS patients. In these five patients, all FI results were confirmed by another technique (RID) and Western blotting, and large genomic deletions were ruled out by MLPA. In addition, FI functional assays showed normal C3b cleavage in all patients. Further research is required to understand this variability. We classified both variants as VUSs (variant of unknown significance).

We completed functional studies on all C3G patients with normal FI levels (p.Lys136Thr, p.Ile244Ser, p.Arg336Gly, p.Asn428Ser) and observed significantly impaired FI function (C3b cleavage) with all cofactors (FH, MCP, sCR1) for only p.Arg336Gly (**Figures 3A–C**). Because maturation of FI requires a proteolytic process that removes four amino acids (Arg-Arg-Lys-Arg) at residues 336-339, FI p.Gly336 circulates as an unprocessed pro-peptide without functional activity (**Figure 3D**). This finding has also been reported for p.Arg339Lys and p.Arg339Glu in aHUS patients (21, 44) and p.Arg339Gln in AMD patients (18, 38, 43). Common to all these mutations is the removal of a positive charged residue from the consensus sequence R-x-K/R-R. Therefore, we would expect individuals carrying these variants to have an identifiable circulating unprocessed single peptide and these variants should be classified as likely pathogenic.

**Figure 4:**
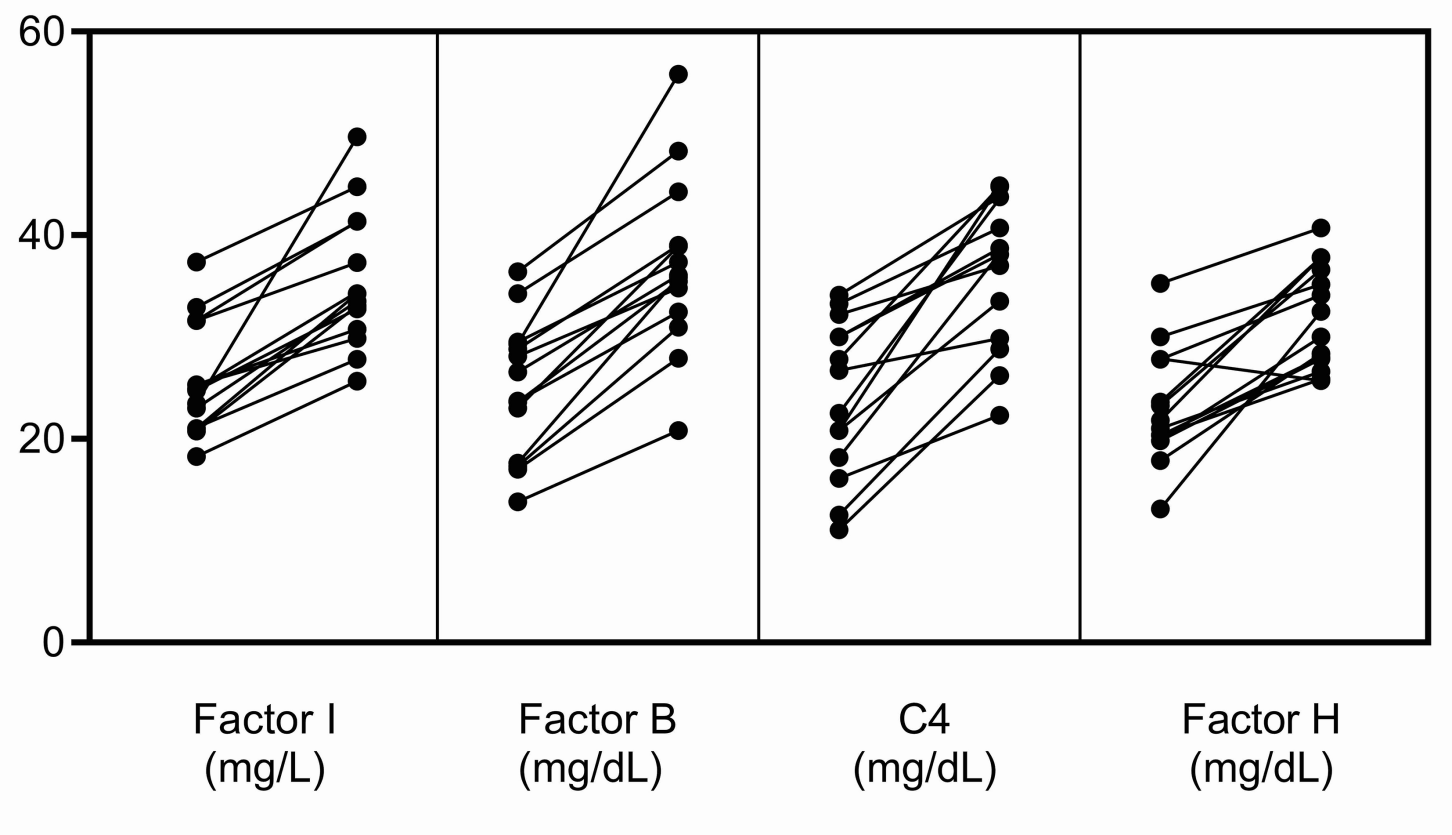
Increases in FI, FB, C4 and FH in the acute phase. These complement proteins are positive acute-phase reactants, and their levels were significantly increased in the acute phase in fourteen patients in our C3G and aHUS registries. The increase in FI does not occur in isolation but rather in conjunction with increases in other complement biomarkers, especially FB, C4 and FH. This pattern of increase was not observed in the patients described in this study (left, complement levels in remission; right, complement levels in the acute phase).

Finally, when measuring FI levels, it should be remembered that as an acute-phase protein its serum concentration increases non-specifically in response to many cytokines (17). In our experience, in the acute phase, FI levels can be elevated ~25% above baseline, however this increase does not occur in isolation but rather in conjunction with an increase in other complement biomarkers, especially FB, C4 and FH (**Figure 4**). To ensure that the impact of a rare hypomorphic allele variant is not masked by the normal allele, serial testing of complement biomarkers is advisable. Anti-C5 therapy with eculizumab does not impact FI levels although it does elevate plasma C5, suppress sC5b-9, and abolish activity of the classical and alternative pathways. And finally, we found that the prevalence of rare *CFI* variants is significantly higher in aHUS as compared to C3G (*P* = 0.0078). While this finding suggests that partial FI deficiency might have less impact on C3G, precisely how a relative deficiency in FI contributes to the underlying pathophysiology of these two diseases requires further study.

In summary, rare variants in *CFI* play a causal role in C3G and aHUS although the clinically observed phenotype is strongly contingent on the associated genetic background. Functional testing should be considered to assess FI activity if a rare *CFI* variant is identified. Our results suggest that the majority of *CFI* missense variants with a MAF < 0.1% will be type 1 variants (~80%), although a small of type 2 variants (~5%) will also be identified.

## Data Availability Statement

The original contributions presented in the study are included in the article/**Supplementary Material**. Further inquiries can be directed to the corresponding author.

## Ethics Statement

The studies involving human participants were reviewed and approved by Institutional Review Board of Carver College of Medicine at the University of Iowa. Written informed consent to participate in this study was provided by the participants’ legal guardian/next of kin.

## Author Contributions

YZ and RS conceived of the study, designed experiments, and wrote the manuscript. RG, NGB, HD, SP, NM, DS, SR, MJ, GP, and AT performed the experiments. CN provided crucial conceptual input. All authors reviewed and approved the manuscript.

## Funding

This work was supported in part by National Institute of Health R01 DK110023.

## Conflict of Interest

The authors declare that the research was conducted in the absence of any commercial or financial relationships that could be construed as a potential conflict of interest.

## Publisher’s Note

All claims expressed in this article are solely those of the authors and do not necessarily represent those of their affiliated organizations, or those of the publisher, the editors and the reviewers. Any product that may be evaluated in this article, or claim that may be made by its manufacturer, is not guaranteed or endorsed by the publisher.

## Supplementary Material

Supplementary Table 1 Biomarker data in patients with aHUS.

Supplementary Table 2 Biomarker data in patients with C3G.
